# Self-assembled adipose-derived mesenchymal stem cells as an extracellular matrix component- and growth factor-enriched filler

**DOI:** 10.3389/fcell.2023.1219739

**Published:** 2023-09-19

**Authors:** Choa Park, Ok-Hee Lee, Jin Ju Park, Jiyoon Yoo, Euna Kwon, Jie-Eun Park, Byeong-Cheol Kang, Dong-Sup Lee, Jaejin Cho

**Affiliations:** ^1^ Department of Dental Regenerative Biotechnology, School of Dentistry, Seoul National University, Seoul, Republic of Korea; ^2^ Dental Research Institute, Seoul National University, Seoul, Republic of Korea; ^3^ Department of Biomedical Sciences, Seoul National University College of Medicine, Seoul, Republic of Korea; ^4^ Department of Experimental Animal Research, Biomedical Research Institute, Seoul National University Hospital, Seoul, Republic of Korea

**Keywords:** mesenchymal stem cell, regenerative medicine, spheroid, microblock, extracellular matrix, growth factor, atrophic scar in addition

## Abstract

The clinical application of mesenchymal stem cells (MSCs) is attracting attention due to their excellent safety, convenient acquisition, multipotency, and trophic activity. The clinical effectiveness of transplanted MSCs is well-known in regenerative and immunomodulatory medicine, but there is a demand for their improved viability and regenerative function after transplantation. In this study, we isolated MSCs from adipose tissue from three human donors and generated uniformly sized MSC spheroids (∼100 µm in diameter) called microblocks (MiBs) for dermal reconstitution. The viability and MSC marker expression of MSCs in MiBs were similar to those of monolayer MSCs. Compared with monolayer MSCs, MiBs produced more extracellular matrix (ECM) components, including type I collagen, fibronectin, and hyaluronic acid, and growth factors such as vascular endothelial growth factor and hepatocyte growth factor. Subcutaneously injected MiBs showed skin volume retaining capacity in mice. These results indicate that MiBs could be applied as regenerative medicine for skin conditions such as atrophic scar by having high ECM and bioactive factor expression.

## 1 Introduction

The skin is a primary tissue that serves as the body’s first line of defense against physical and environmental assaults. When the skin is injured, the wound healing process is activated, which can result in scar formation. Ideally, the wound healing process leads to nearly scarless skin. Unfortunately, some individuals may experience excessive fibrosis or atrophy, leading to keloidal, hypertrophic, or atrophic scars (Tsao et al., 2002). Atrophic scarring produces a depressed scar, unlike keloidal and hypertrophic scars that involve tissue outgrowth. The topographical depression of atrophic scars is thought to result from inadequate compensation of dermal collagen and connective tissue after injury (Weiss et al., 2010). Atrophic scarring can occur due to various reasons, including scleroderma (Roh et al., 2008), stretch marks during pregnancy (Lokhande and Mysore, 2019), acne (Fabbrocini et al., 2010), surgery, and accidents (Weiss et al., 2010). Pathological scars can significantly reduce the quality of life and cause psychological stress, particularly when located in visible areas of the body. Therefore, treatment of scars can help improve the quality of life.

Several treatments have been used to improve scar appearance, including subcision, lasers, and filler injection (Tsao et al., 2002). Dermal fillers such as hyaluronic acid (HA), collagen, poly-L-lactic acid, calcium hydroxylapatite, and polymethylmethacrylate, are widely used for scar management (Tam et al., 2022). However, HA and collagen are short-lasting fillers that persist for several months at most. In contrast, permanent fillers such as polymethylmethacrylate and silicone are longer-lasting but have higher risks of complications such as granulomas, silicone embolism syndrome, and nodule formation (Cheng et al., 2016). To overcome the limitations of artificial fillers, cell-based therapies, particularly those using stem cells, have emerged as a novel approach (Ahn and Mao, 2010; Dos Santos Machado et al., 2020). For instance, adding mesenchymal stem cells (MSCs) to autologous fat grafts increase the survival rate of the grafts in a mouse fat transplantation model (Zhu et al., 2010). In a pilot clinical study, intradermal MSCs injection improve atrophic acne scars by enhancing skin hydration, tightness, and texture (Ibrahim et al., 2015). To enhance the retention of transplanted MSCs, they can be encapsulated in biocompatible hydrogels before injection. Self-cross-linkable hydrogels using thiol-functionalized HA can effectively deliver MSCs with improved viability and efficacy after implantation under the skin (Lee et al., 2022). However, encapsulation of stem cells can be challenging as the stiffness, degradation rate, and pore size of hydrogels can impact stem cell outcomes such as differentiation (Tsou et al., 2016). To address these challenges, it is crucial to develop a MSC delivery method in which cells reside within a purely cell-derived extracellular matrix (ECM) devoid of artificial components. In this regard, we have generated uniformly sized MSC aggregates called microblocks (MiBs) that are rich in ECM components and growth factors. Our studies have demonstrated the potential for long-term volumetric retention of MSCs after subcutaneous implantation of MiBs, making them a promising dermal filler for the treatment of atrophic scars.

## 2 Materials and methods

### 2.1 Cell culture

Adipose-derived MSCs (ADMSCs) were obtained from adipose tissue from three healthy human donors as described previously (Ganbold et al., 2019). All experimental procedures in this study were approved by the Institutional Review Board of the School of Dentistry, Seoul National University (IRB No. S-D20150019). In brief, lipid tissue was collected from liposuction specimens, digested with 0.1% collagenase I (Gibco, Carlsbad, CA) in Hanks’ balanced salt solution (HyClone Laboratories, Logan, UT), and passed through a 100-µm strainer (BD Falcon, Franklin Lakes, NJ). Cells were resuspended in high-glucose Dulbecco’s modified Eagle’s medium (HyClone Laboratories) containing 10% fetal bovine serum (HyClone Laboratories). All cultures were maintained in a humidified incubator at 37°C and 5% CO_2_. Cells were passaged at ∼70% confluence, and cells at passages 3 to 7 were used. To generate MiBs, ADMSCs were seeded on AggreWell™400 plates (Stem Cell Technology, Cambridge, MA) and cultured for the 1, 2, 3, or 7 days to form MSC spheroids. To collect spheroids of uniform size, spheroids were passed through double-stacked cell strainers with pore sizes of 70 μm and 300 μm. MiBs were imaged using the JuLi stage system (NanoEnTek, Seoul, Korea), and MiB diameter was measured using ImageJ software (National Institutes of Health, version 1.53j, https://imagej.nih.gov/ij/download.html).

### 2.2 Proliferation

The cumulative population doubling level (CPDL) was calculated at each passage using the following equation:

$$X=\left[\log_{10}\left(N_{H}\right)–\log_{10}\left(N_{I}\right)\right]/\log_{10}\left(2\right)$$

where *N*_*I*_ is the initial cell number, *N*_*H*_ is the harvest cell number, and *X* is population doubling. To determine the CPDL, the calculated population doubling increase was added to the population doubling level of the previous passage (Cristofalo et al., 1998). Population doubling time (PDT) was calculated using the following equation:

$$PDT=T\times \log2/\left(\log N_{H}–\log N_{I}\right)$$

where *T* is culture time (Zhan et al., 2019).

### 2.3 Flow cytometry

Single cells detached or dissociated by TrypLE (Gibco) treatment were stained with fluorescence-conjugated antibodies against CD29 (BD Bioscience, Franklin Lakes, NJ), CD44 (BD Bioscience), CD73 (BD Bioscience), CD90 (BD Bioscience), CD105 (eBioscience, San Diego, CA), CD31 (BD Bioscience), CD45 (eBioscience), CD79a (eBioscience), CD117 (eBioscience), or human leukocyte antigen DR (HLA-DR; BD Bioscience). Isotype-matched control antibodies were used as negative controls for staining. After washing with phosphate-buffered saline, flow cytometric analysis was conducted with FACSLyric (BD Bioscience), and data were analyzed using FACSuite (BD Bioscience).

### 2.4 Adipogenic, osteogenic, and chondrogenic differentiation

For adipogenic differentiation, ADMSCs were differentiated using growth medium containing 0.5 mM isobutylmethylxanthine (Sigma-Aldrich, St. Louis, MO), 10 μg/mL insulin (Sigma-Aldrich), 100 nM dexamethasone (DEXA; Sigma-Aldrich), and 100 μM indomethacin (Sigma-Aldrich) for 2 weeks. Adipogenic differentiation was confirmed by staining cells with Oil Red O (Sigma-Aldrich). For osteogenic differentiation, ADMSCs were incubated in α-minimum essential medium (α-MEM; Welgene, Daegu, Korea) containing 10% fetal bovine serum, 1% amino acids, 10 mM β-glycerophosphate (Sigma-Aldrich), 50 μg/mL ascorbic acid (Sigma-Aldrich), 10 nM DEXA, and 1 mM dibutyryl cyclic adenosine monophosphate (Sigma-Aldrich) for 2 weeks. Fixed cells were stained with Alizarin Red S (Sigma-Aldrich). For chondrogenic differentiation, cells were pellet-cultured in chondrogenic differentiation medium, which was α-MEM containing 1% amino acids, 50 ng/mL ascorbic acid, 100 nM DEXA, 10 ng/mL transforming growth factor-β3 (R&D Systems, Minneapolis, MN), 1× Insulin-Transferrin-Selenium Supplement (Gibco), and 40 μg/mL L-proline (Sigma-Aldrich) for 3 weeks. Fixed pellets were embedded in optimal cutting temperature compound medium (Sakura Finetek, Torrance, CA). Cryostat sections (8-μm thick) were stained with Alcian Blue (Sigma-Aldrich).

### 2.5 Cell viability

Cell viability was assessed using a 3-(4,5-dimethyl-2-thiazolyl)-2,5-diphenyl-2H-tetrazolium bromide (MTT) Cell Proliferation Assay kit (R&D Systems) and LIVE/DEAD™ Viability/Cytotoxicity Kit (Invitrogen, Grand Island, NY) according to the manufacturer’s instructions.

### 2.6 Scanning electron microscopy

Samples were post-fixed with 1% osmium tetroxide on ice for 1 h. After dehydration, MiBs were chemically dried using hexamethyldisilazane. Samples were coated with a layer of platinum using Q150TS (Quorum Technologies, Lewes, UK). Scanning electron microscopy (SEM) was performed using Apreo S (Thermo Fisher Scientific) with a voltage of 10 kV.

### 2.7 Immunostaining

MiBs were fixed and blocked using an Image-iT Fixation/Permeabilization Kit (Invitrogen) according to the manufacturer’s instructions. MiBs were stained with anti-CD73 (1:500; ab175396; Abcam, Cambridge, MA), anti-CD90 (1:500; ab133350; Abcam), anti-CD105 (1:500; ab11414; Abcam), or anti-type I collagen (1:500; ab34710; Abcam) antibodies. To identify implanted MiBs in skin tissues, deparaffinized sections were stained with STEM 101 antibody (1:50; Y40400; Takara, Shiga, Japan). For immunofluorescence, primary antibodies were visualized by incubation with Alexa 488- or Alexa 594-conjugated antibodies (1:2000; Invitrogen). Nuclei were stained with 4′,6-diamidino-2-phenylindole (DAPI; Sigma-Aldrich). To stain blood vessels, consecutive sections of skin tissues were subjected to staining with anti-CD31 (1:100; ab28364; Abcam) or anti-XRCC5 (1:200; #2180; Cell Signaling Technology, Denvers, MA, United States) antibodies using the Discovery XT automated immunohistochemistry stainer (Ventana Medical Systems, Inc., Tucson, AZ, United States). Detection was performed using the Ventana ChromoMap Kit (Ventana Medical Systems), and the sections were counterstained with Hematoxylin and Bluing reagent. MiB paraffin sections were deparaffinized and incubated with anti-type I collagen antibody (1:500; ab34710; Abcam) and processed using a VECTASTAIN Elite ABC-HRP Kit (Vector Laboratories, CA, United States). Images were captured using a Nikon DS-Ri2 camera attached to a Nikon Eclipse Ti microscope (Nikon, Tokyo, Japan) or LSM700 confocal laser scanning microscope (Carl Zeiss, Oberkochen, Germany).

### 2.8 Masson’s trichrome staining

MiBs and tissues were fixed with 4% paraformaldehyde, embedded in paraffin, and sectioned to a thickness of 4 μm. Deparaffinized sections were stained using a Trichrome Staining Kit (Hoffman-La Roche, Basel, Switzerland) according to the manufacturer’s instructions.

### 2.9 Enzyme-linked immunosorbent assay

ADMSCs or MiBs were digested in Mammalian Protein Extraction Reagent (Thermo Scientific Inc., Waltham, MA) with 1 mM phenylmethanesulfonyl fluoride (Sigma) and Halt™ Protease Inhibitor Cocktail (Thermo Scientific). Samples were transferred into a gentleMACS Tube (Miltenyi Biotec, Bergisch Gladbach, Germany) and homogenized with a gentleMACS dissociator (Milternyi Biotec). After being centrifuged for 20 min at 16,000 × g at 4°C, the level of type I C-terminal collagen propeptide (CICP) in the supernatant was measured using MicroVue CICP EIA (Quidel, San Diego, CA), and the concentration of HA was analyzed using an enzyme-linked immunosorbent assay (ELISA) kit from MyBioSource (San Diego, CA, United States) according to the manufacturer’s instructions.

### 2.10 Glycosaminoglycan assay

Glycosaminoglycan (GAG) concentration and total cellular DNA content were measured using GAG Assay Blyscan™ (Biocolor, Carrickfergus, UK) and Quant-iT™ PicoGreen™ dsDNA Assay kits (Invitrogen), respectively. The level of GAG expression was normalized to the amount of DNA content.

### 2.11 Reverse transcription polymerase chain reaction

Total RNA was isolated using TRI reagent (Invitrogen) with chloroform (Sigma) according to the manufacturer’s instructions. cDNA was synthesized from 1 µg total RNA using Prime Script RT Master Mix (Takara, Tokyo, Japan). For quantitative real-time reverse transcription polymerase chain reaction (qRT-PCR) analysis, standard curves were created for each target gene primer set using known quantities of total cDNA from other cells. PCR reactions were performed in triplicate using 2× qPCRBIO SyGreen Mix Hi-ROX (PCR Biosystems, Wayne, PA) on a QuantStudio 3 Real-time PCR system (Applied Biosystems, Foster City, CA). For conventional RT-PCR, PCR reactions were performed using AccuPower PCR PreMix (Bioneer) on a PCR machine (Bio-Rad Laboratories, Hercules, CA, United States). The PCR products were analyzed by agarose gel electrophoresis. The primer sets in this study are listed in Supplementary Table S1, S2. QuantiTect primers for COL1A1, COL3A1, ELN, and FN were purchased from Qiagen (Hilden, Germany).

### 2.12 Volumetric analysis

Seven-week-old non-obese diabetic SCID γ (NSG) mice (NOD.Cg-Prkdc^scid^ Il2rg ^tm 1Wjl^/SzJ) were purchased from Orient Bio (Seongnam, Korea). Mice were given *ad libitum* access to a commercial rodent diet and tap water. All experimental procedures involving mice were approved by the Institutional Animal Care and Use Committee of the Biomedical Research Institute at Seoul National University Hospital (IACUC No. SNU-171107-4-3). Twenty-four mice were utilized to investigate volume retention following the injection of MiBs. The dorsal skin of each mouse was subcutaneously injected with four different substances: (1) normal saline, (2) vehicle control (10-fold diluted HA (dHA) filler; YVOIRE Classic Plus; LG Chem, Seoul, Korea), (3) low-dose MiB (500 MiBs), or (4) high-dose MiB (5,000 MiBs). Two left-sided and two right-sided regions of the dorsal skin were chosen for the injection. For assessment of retained volume, the length, width, and height of the injected volume were measured weekly for 12 weeks using a caliper. Volume was calculated using the following equation (Hillel et al., 2012):

$$Volume=4/3\;x\;\pi \;x\;\left(1/2\;x\;length\right)\;x\;\left(1/2\;x\;width\right)\;x\;\left(1/2\;x\;height\right)$$

### 2.13 PCR amplification of human Alu-sx and mouse c-mos sequences

Mice were sacrificed 4 or 8 weeks after MiB transplantation. DNA was extracted from skin samples using an AccuPrep Genomic DNA Extraction Kit (Bioneer, Daejeon, Korea). DNA was amplified using AccuPower PCR PreMiX (Bioneer) with primer sets for the human Alu sequence (forward, 5′-GGC​GCG​GTG​GCT​CAC​G-3′ and reverse, 5′-TTT​TTT​GAG​ACG​GAG​TCT​CGC​TC-3′) and mouse c-mos sequence (forward, 5′-GAA​TTC​AGA​TTT​GTG​CAT​ACA​CAG​TGA​CT-3′ and reverse, 5′-AAC​ATT​TTT​CGG​GAA​TAA​AAG​TTG​AGT-3′). PCR conditions were 94°C for 5 min followed by 35 cycles of 94°C for 30 s, 58°C for 15 s, and 72°C for 30 s, followed by a final step of 72°C for 5 min. PCR products were confirmed by electrophoresis through 1% agarose gel. MiBs were used as positive controls for the Alu sequence, and non-transplanted mouse skin was used as a positive control for the c-mos sequence.

### 2.14 Statistical analysis

After confirming that the data followed a normal distribution, comparisons between two groups were performed using independent samples t-tests. In the animal study, comparisons between groups were performed using one-way ANOVA followed by Dunnett’s and Tukey’s Honestly Significant Difference tests. The data is presented as mean ± standard deviation (SD). Statistical analyses were performed using SPSS version 26 (IBM, Armonk, NY, United States). Significant differences are denoted as *p < 0.05, **p < 0.01, and ***p < 0.001.

## 3 Results

### 3.1 Generation and characterization of MSC spheroid MiBs

**FIGURE 1:**
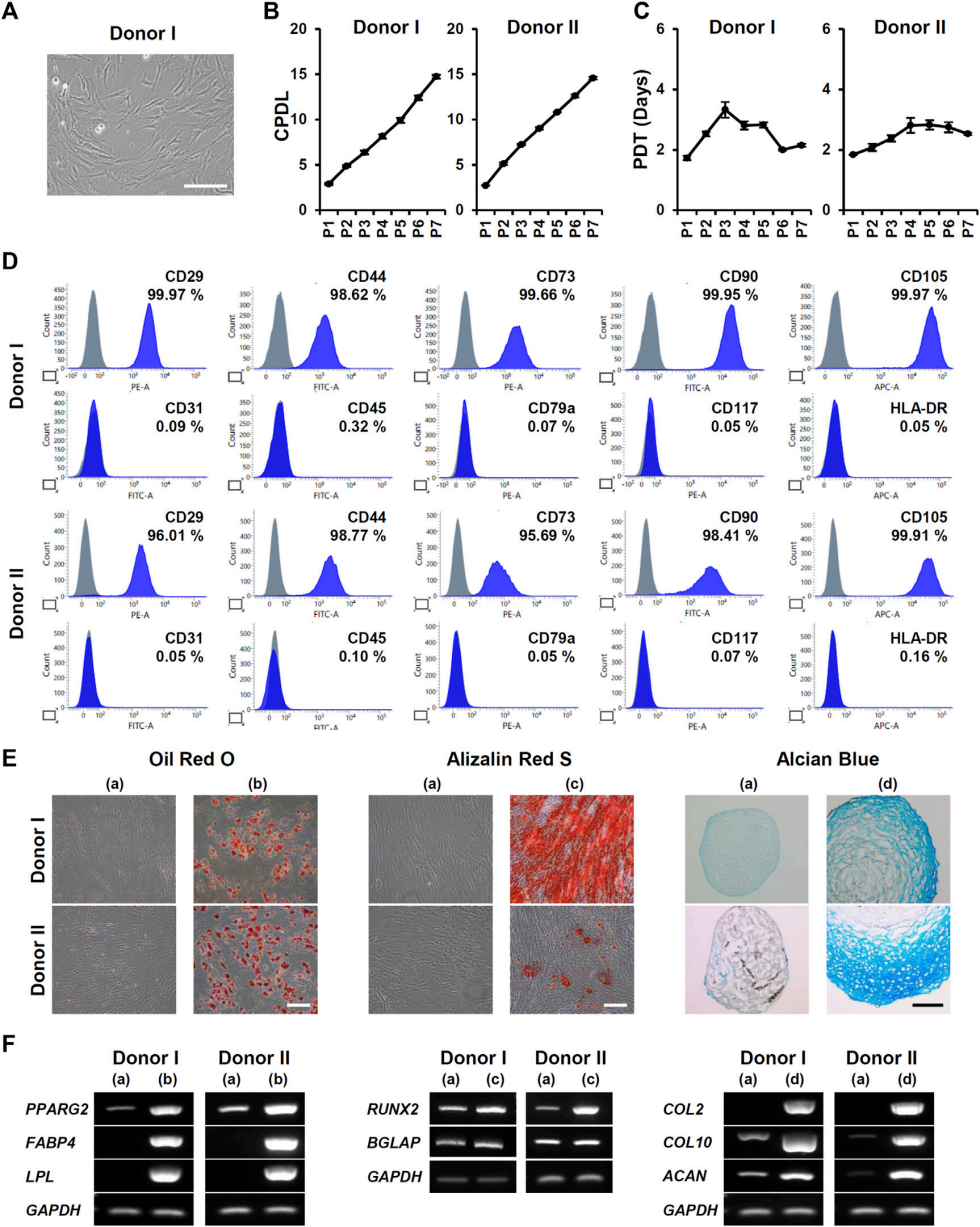
Isolation and characterization of ADMSCs from donors I and II. **(A)** Representative microscopic image of ADMSCs in monolayer culture. Cells were isolated from donor I. Scale bar, 200 μm. **(B)** The cumulative population doubling level (CPDL) and **(C)** population doubling time (PDT) of ADMSCs were measured at each passage (*n* = 6). **(D)** Expression of positive markers (CD29, CD44, CD73, CD90, and CD105) and negative markers (CD31, CD45, CD79a, CD117, and HLA-DR) of ADMSCs were analyzed using flow cytometry. **(E)** The differentiation potential of ADMSCs into adipocytes (b), osteocytes (c), and chondrocytes (d) was compared with that of undifferentiated cells (a) by Oil Red O, Alizarin Red S, and Alcian Blue staining, respectively. Scale bar, 200 μm. **(F)** Gene expression of differentiation markers was analyzed by RT-PCR. Total RNA was isolated from undifferentiated ADMSCs (a), and differentiated adipocytes (b), osteocytes (c), and chondrocytes (d).

To study the effects of 3D culture on MSC characterization, we obtained ADMSCs from three healthy donors (donner I, II, and III). The stemness of isolated ADMSCs was verified by proliferation ability, MSC marker expression, and tri-lineage differentiation. Cells showed a fibroblast-like spindle shape in monolayer culture (Figure 1A). Proliferative capacity was actively maintained through seven passages (Figures 1B, C). More than 95% of ADMSCs were positive for CD29, CD44, CD73, CD90, and CD105 expression, and less than 2% were positive for CD31, CD45, CD79a, CD117, and HLA-DR expression (Figure 1D; Supplementary Figure S1A). Adipogenic, osteogenic, and chondrogenic differentiation of ADMSCs were confirmed by Oil Red O, Alizarin Red S, and Alcian Blue staining, respectively (Figure 1E; Supplementary Figure S1B). RT-PCR analysis also showed an increase in the expression of differentiation-related genes: *PPARG2*, *FABP4*, and *LPL* for adipogenesis; *RUNX2* and *BGLAP* for osteogenesis; and *COL2*, *COL10*, and *ACAN* for chondrogenesis (Figure 1F; Supplementary Figure S1C).

**FIGURE 2:**
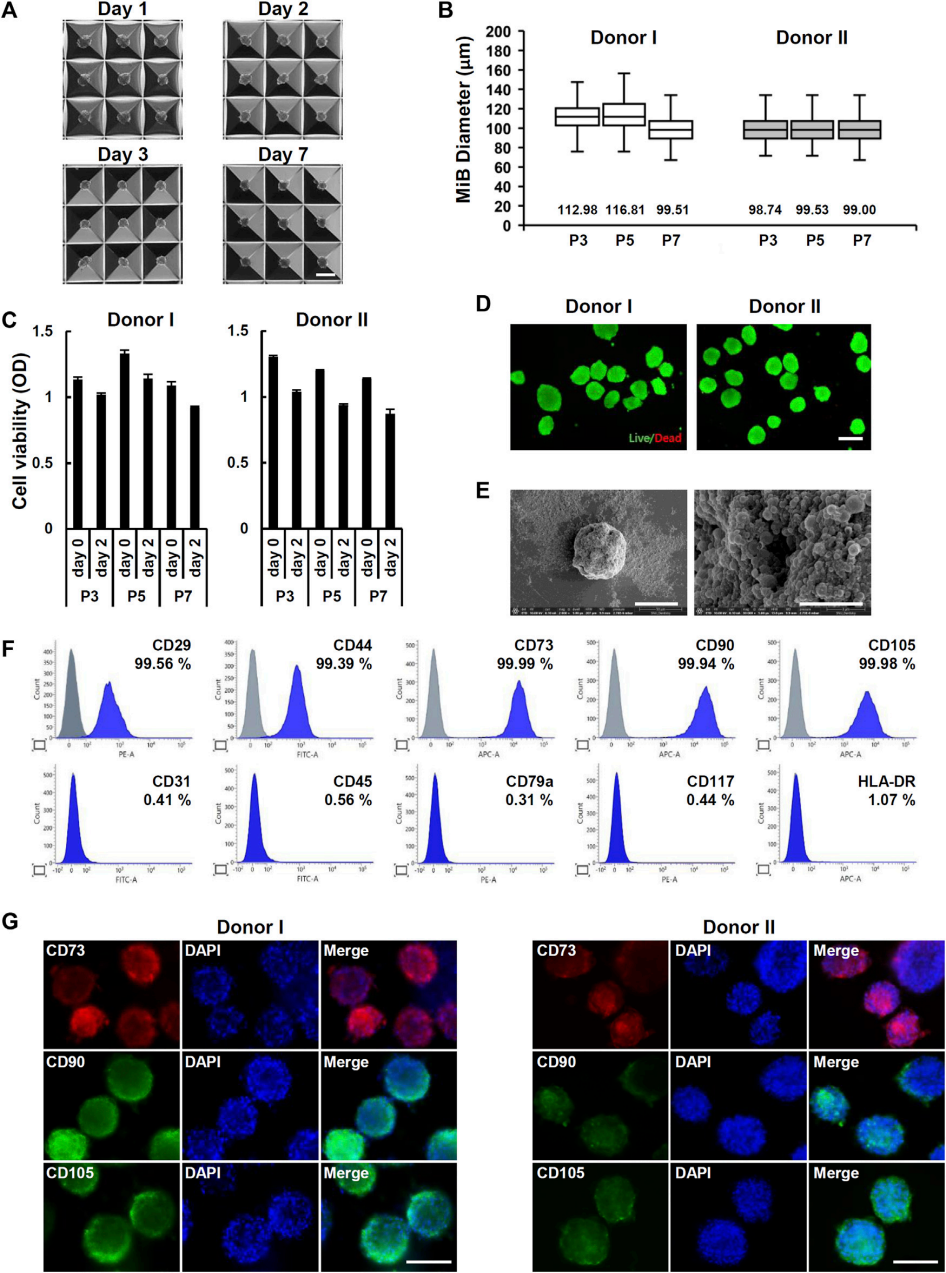
Generation of MiBs using Aggrewell™400 plates. **(A)** Images of uniformly sized MiBs grown inside microwells for 1, 2, 3, and 7 days were taken at ×40 magnification. MiBs were generated using ADMSCs isolated from donor I. Scale bar, 200 μm. **(B)** The diameters of MiBs cultured for 2 days in AggreWell™400 plates were measured using ImageJ software. MiBs were generated using MSCs in passages 3, 5, and 7. Over 130 MiBs were analyzed per passage to determine their diameter. Data represent the mean MiB diameter ± SD. **(C)** The viability of MiBs was assessed using the MTT assay kit. MiBs were prepared at passages 3, 5, and 7, and their viability was compared to monolayer MSCs (day 0) (*n* = 4). **(D)** Analysis of live (green) and dead (red) cells in MiBs at day 2 was performed using a LIVE/DEAD kit. Scale bar, 200 μm. **(E)** SEM analysis showed small aggregates around MiBs at day 2 (2,000×) (left panel, Scale bar, 50 μm). Higher magnification showed the formation of small membrane vesicles on MiB surfaces (30,000×) (right panel, Scale bar, 5 μm). **(F)** MiBs were cultured in AggreWell™400 plates for 2 days, and expression of MSC surface markers was confirmed using flow cytometry. Positive (CD29, CD44, CD73, CD90, and CD105) and negative (CD31, CD45, CD79a, CD117, and HLA-DR) markers of human MSCs were analyzed. **(G)** Immunofluorescence staining showed CD73, CD90, and CD105 expression in MiBs at day 2. DAPI was used to stain nuclei. Images were taken at ×400 magnification. Scale bar, 100 μm.

To form ADMSC spheroids called MiBs, ADMSCs were seeded on AggreWell™400 plates. The MiBs that formed in microwells were ∼100 µm in diameter. The size of MiBs did not increase across 7 days of incubation (Figure 2A). The size and cell viability of MiBs generated with ADMSC at passages 3, 5, and 7 showed no significant differences (Figures 2B, C). According to live and dead cell staining, most cells constituting MiBs were viable (Figure 2D). To analyze the proliferation of cells within the MiBs, we measured the DNA content of MiBs cultured for 1, 2, 3, and 7 days. However, no significant cell proliferation was detected, as shown in Supplementary Figure S2. Morphological analysis using SEM showed the compact and spherical structure of MiBs (Figure 2E). MiBs displayed a rough surface and many blebs. To examine whether the cells in MiBs retained MSC characteristics, MiBs were dissociated into single cells, and the expression of MSC surface markers was analyzed using flow cytometry. MiBs showed positive expression of MSC markers such as CD29, CD44, CD73, CD90, and CD105 but negative expression of CD31, CD45, CD79a, CD117, and HLA-DR (Figure 2F). Immunofluorescence staining further confirmed that MiBs showed high expression of the MSC markers CD73, CD90, and CD105 (Figure 2G). These results indicate that MiBs retained MSC properties.

### 3.2 Enhanced expression of ECM in MiBs

**FIGURE 3:**
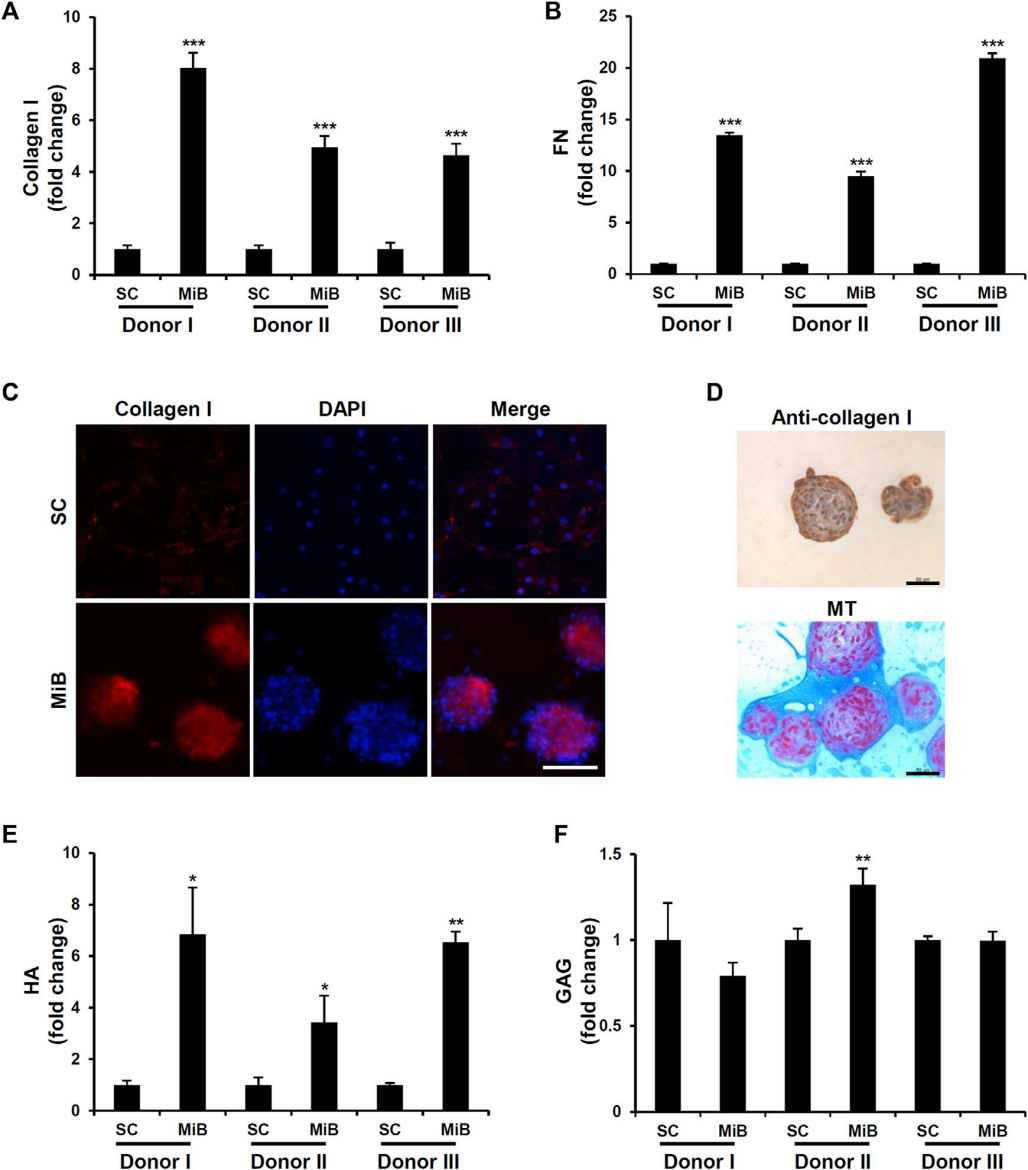
Enhanced ECM expression in MiBs on day 2. **(A)** Type I collagen expression was compared between MiBs and monolayer MSCs (SC). Type I collagen was measured by ELISA (*n* = 3). **(B)** Fibronectin (FN) expression was measured in monolayer MSCs and MiBs using ELISA (*n* = 3). **(C)** Immunofluorescence staining using anti-type I collagen antibody confirmed higher expression of type I collagen in MiBs than in monolayer MSCs. Scale bar, 100 μm. **(D)** Immunohistochemistry (brown) and Masson’s trichrome staining (blue) showed enrichment of collagen expression in MiBs. Scale bar, 50 μm **(E)** HA was increased in MiBs. Relative HA level was normalized by total protein level (*n* = 3). **(F)** GAG expression was measured by a GAG assay kit and normalized by DNA content (*n* = 3). Data represent the mean of fold change ± SD. Statistical analysis was conducted using independent samples t-test. * *p* < 0.05, ** *p* < 0.01, *** *p* < 0.001 vs. monolayer MSCs (SC).

MiBs are a 3D cell culture that creates an *in vivo–*like environment involving cell-cell and cell-ECM interactions. To examine whether cells in MiBs differentially expressed fibrous proteins such as collagen type I and III, elastin, and fibronectin, real-time RT-PCR analysis was performed. The analysis indicated that mRNA expression of collagen type I and fibronectin was significantly increased in MiBs compared with 2D-cultured ADMSCs (Supplementary Figure S3). Consistent with these RT-PCR results, the protein levels of collagen type I and fibronectin were increased in all MiBs derived from the three donors (Figures 3A, B). As collagen type I is the major structural component of native ECM in living tissue and the common type in skin, tendon, vasculature, and organs, we further analyzed the expression of collagen type I using immunostaining. Collagen type I proteins were highly detected in MiBs (Figure 3C). However, monolayer ADMSCs were not stained with anti-collagen type I antibodies due to very low detectable levels of the protein in cells. Similar to the results of immunofluorescence staining, MiBs clearly expressed collagen type I in immunohistochemical analysis and Masson’s trichrome staining (Figure 3D). As other major ECM components, HA and GAG are also important for forming structural networks and retaining moisture in the skin. We measured HA and GAG to examine whether their levels were enhanced in MiBs. HA was abundant in MiBs compared with ADMSCs in 2D culture, but GAG levels were similar between MiBs and ADMSCs (Figures 3E, F).

### 3.3 Expression of growth factors in MiBs

**FIGURE 4:**
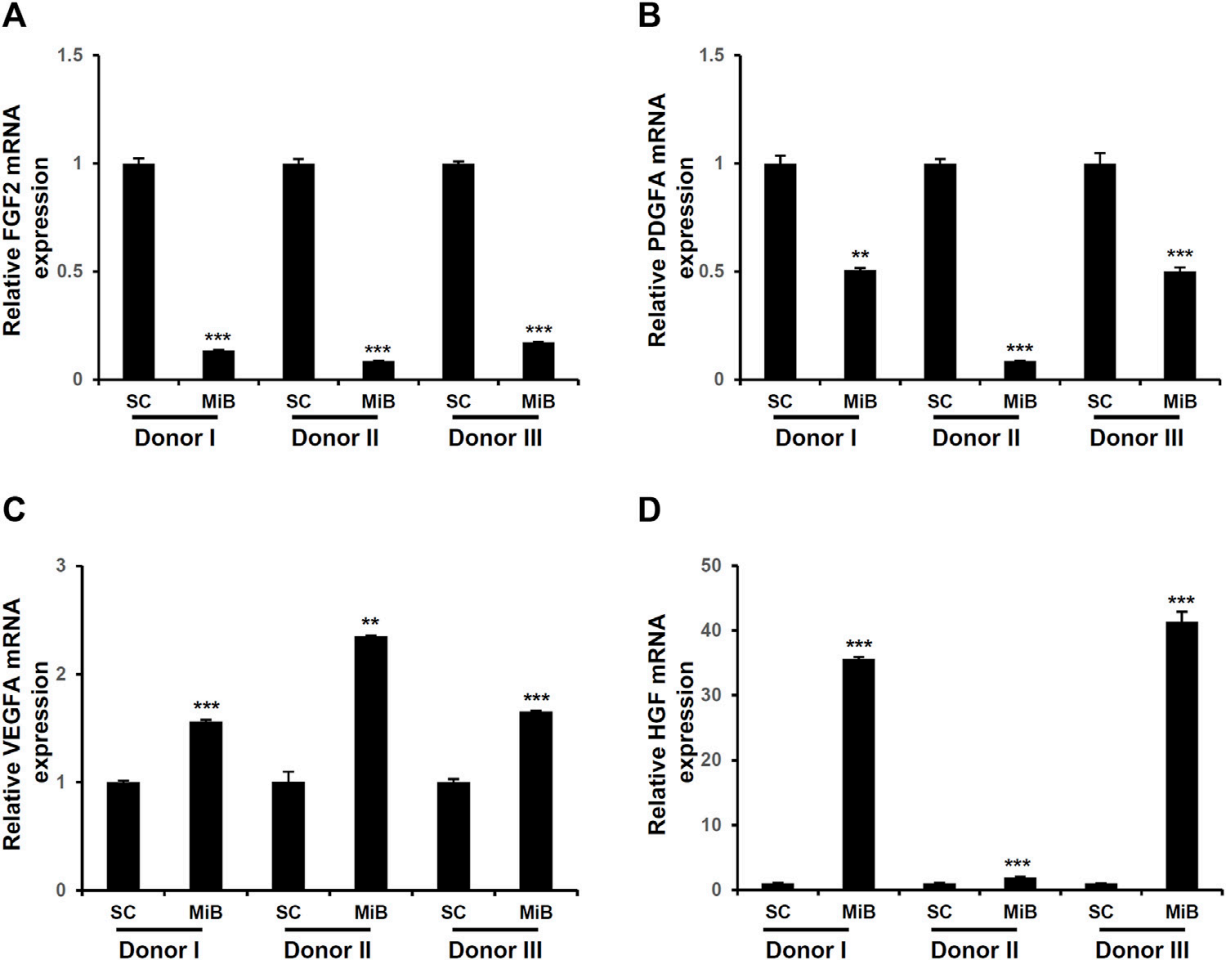
Expression of growth factors in MiBs on day 2. Expression of FGF2 **(A)**, PDGFA **(B)**, VEGFA **(C)**, and HGF **(D)** were analyzed by real-time PCR. Relative expression was compared with monolayer MSCs (SCs) (*n* = 3). Data represent the mean ± SD. Statistical analysis was conducted using independent samples t-test. **p < 0.01, ***p < 0.001 vs. monolayer MSCs (SC).

To determine the expression of growth factors in MiBs, qRT-PCR was performed. The expression of fibroblast growth factor 2 (FGF2) and platelet-derived growth factor subunit A (PDGFA) were decreased in MiBs on day 2 (Figure 4). Also, the expression of vascular endothelial growth factor A (VEGFA) and hepatocyte growth factor (HGF) were increased in MiBs compared with ADMSCs in 2D culture (Figure 4). In particular, the degree of increase in HGF expression in MiBs was dependent on the adipose tissue donor. MiBs from donor I and III showed more than a 30-fold increase in HGF expression compared with ADMSCs in 2D culture, and MiBs from donor II also showed a 2-fold increase in HGF expression (Figure 4).

### 3.4 Volume maintenance and survival of MiBs *in vivo*

**FIGURE 5:**
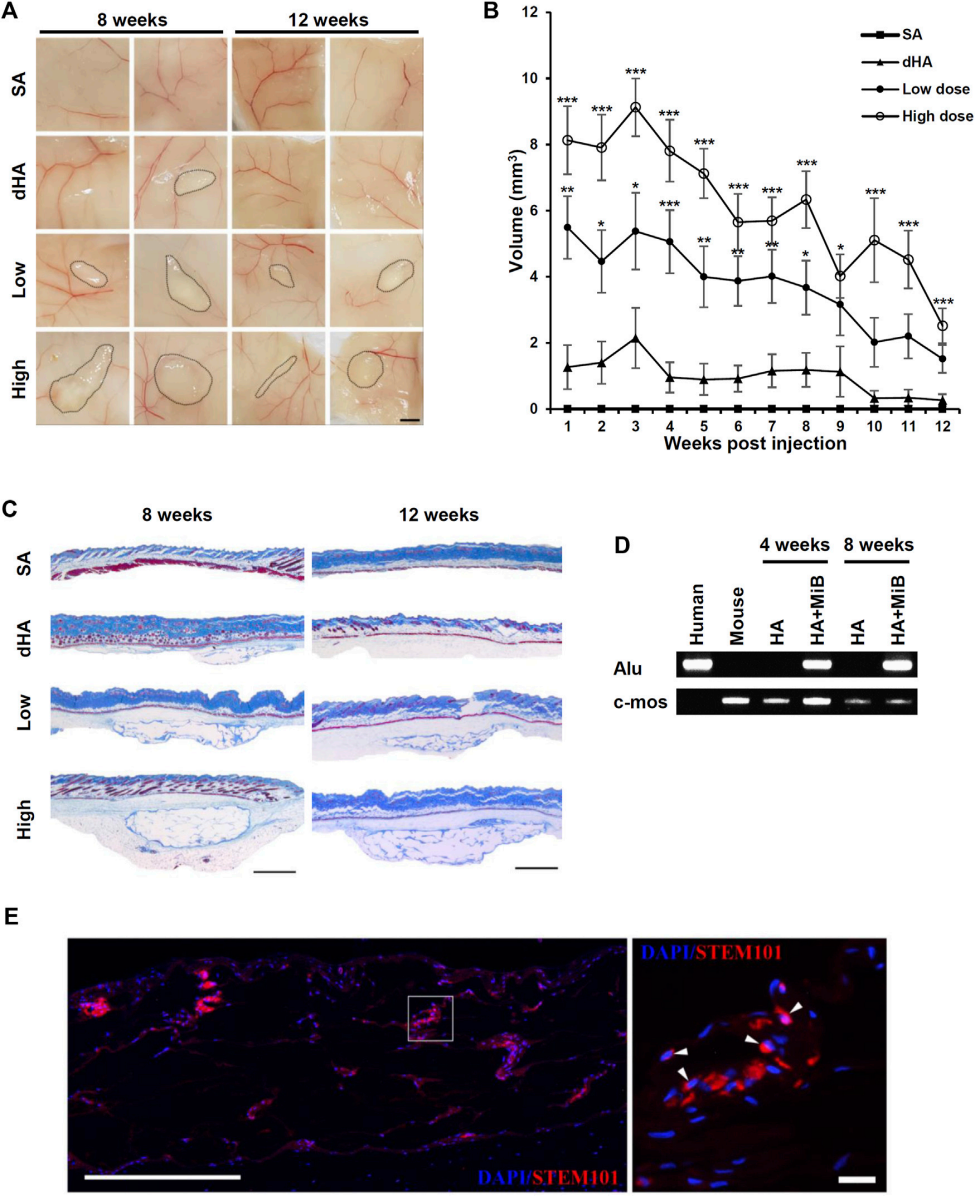
Sustained filling effect of subcutaneously injected MiBs. Low-dose (500 MiB) and high-dose (5,000 MiB) MiBs were subcutaneously injected into NSG mice. Saline (SA) and diluted HA (dHA) were used as controls. **(A)** Stereoscopic images of injected MiBs under the skin were taken after surgical removal of injected sites. The retained volumes were confirmed 8 and 12 weeks post-transplantation of MiBs. Scale bar, 200 μm. **(B)** The retention volume of MiBs, which were subcutaneously injected into NSG mice, was measured weekly for up to 12 weeks post-transplantation. From weeks 1 to 8, the volume was measured in 24 mice, and subsequently, 12 mice were sacrificed for histological examination. From weeks 9 to 12, the volume was measured in 12 mice. Data represent the mean ± SD. Statistical analysis was conducted using one-way ANOVA followed by Dunnett’s and Tukey’s Honestly Significant Difference tests. *p < 0.05, **p < 0.01, ***p < 0.001 vs. dHA. **(C)** Masson’s trichrome-stained images of skin tissue sections from different experimental groups. Scale bar, 1 mm. **(D)** Human Alu-sx and mouse c-mos sequences were detected in skin tissue from the MiB-injected group by PCR. **(E)** Transplanted cells are shown in merged images. Skin tissue sections were stained by STEM 101 (red). DAPI (blue) was used for nuclear staining. The white arrows indicate representative STEM 101-positive cells. Scale bar, 500 μm in the left image and 20 μm in the right image.

To evaluate the volume retention of MiBs under the skin, we used NSG mice, which were severely immunodeficient due to deficiencies in T, B, and functional NK cells. This choice of mice allowed for adequate testing of human cell engraftment in mouse model. Moreover, the pronounced immunodeficiency of NSG mice is known to be suitable for conducting long-term persistence studies of engrafted cells compared to other immunocompromised mouse models (Racki et al., 2010). Saline, dHA filler, low-dose MiBs (500 MiBs), and high-dose MiBs (5,000 MiBs) were subcutaneously injected in the dorsal region of NSG mice. The retained volume was measured until 12 weeks post-transplantation. The volumes of low- and high-dose MiBs were retained until 4 weeks after injection. Although both doses of MiBs decreased in volume starting 5 weeks post-injection (Figure 5A), MiB-injected groups displayed measurable volumes across the 12-week period. In addition, the volume of high-dose MiBs was maintained significantly better than the volume of dHA 12 weeks after injection (Figure 5B). Histological analysis indicated that unlike dHA transplantation, which disappeared 8 weeks post-transplantation, MiBs remained in the subcutaneous area of mice, and multi-septum structures were observed in low- and high-dose MiB groups (Figure 5C). To confirm the survival of transplanted MiBs, PCR using the human-specific Alu sequence and silver-enhanced *in situ* hybridization for HER2 were performed. PCR showed that the human-specific Alu sequence, which is expressed only in human cells, was expressed in tissue samples from mice 4 and 8 weeks after MiB transplantation (Figure 5D). Immunofluorescence staining using STEM 101, which specifically stains the nuclei of human cells, also confirmed the successful engraftment and survival of transplanted cells in a injected area (Figure 5F).

To investigate the correlation between MiBs retention and vascularization, we conducted immunostaining on the skin tissues injected with MiBs using an anti-CD31 antibody capable of reacting with both human and mouse endothelial cells. The immunostaining demonstrated the integration of microvessels at the engraftment site. Given the multilineage differentiation potential of MSCs, including their ability to differentiate into endothelial cells (Ikhapoh et al., 2015; Khaki et al., 2018), it is crucial to discern whether the endothelial cells originated from mouse neovascularization or human MSC differentiation. To address this, we stained consecutive sections of the tissue sample using an anti-human XRCC5 antibody enabling specific detection of human nuclei. Consistent with the staining results obtained using STEM101, we observed the presence of human MSCs derived from the MiBs in the injected tissues. Notably, the CD31-positive microvessels did not exhibit detectable staining with the anti-human XRCC5 antibody (Supplementary Figure S4). This finding suggests that the microvessels found in the injected MiBs originated from neighboring mouse vessels rather than differentiation of the human MSCs.

## 4 Discussion

MSCs have attracted attention for their therapeutic potential in regenerative medicine. MSCs possess the ability to migrate to damaged tissue and act as a “drug store” by serving as a reservoir of bioactive molecules for tissue repair (Ullah et al., 2019). Therefore, the therapeutic application of MSCs to treat skin damage has also been intensively investigated. In fact, many studies demonstrate the positive effects of MSCs on wound healing and scar formation by means of controlling inflammation, proliferation, and remodeling of injured skin tissue. In this study, we report the enhanced expression of ECM and growth factors in MSC spheroids, called MiBs. *In vivo* experiments in which MiBs were subcutaneously injected into mice showed their high volume retention capacity, suggesting that MiBs may serve as a dermal filler for the treatment of depressed scars such as atrophic scars.

Skin rejuvenation and regeneration strategies targeting skin aging and wrinkle formation using MSCs are being carried out worldwide. In particular, autologous ADMSCs are promising therapeutic candidates for skin regeneration due to their immune tolerance, growth factor secretion, angiogenic effects, and anti-scarring effects (Zhang et al., 2020). Despite the therapeutic potential of ADMSCs, the 2D-cultured MSC approach for skin rejuvenation shows mild efficacy in various clinical trials (Kouroupis and Correa, 2021). To enhance the therapeutic capability of ADMSCs, a 3D culture method for MSCs was developed (Cesarz and Tamama, 2016; Kouroupis and Correa, 2021). The 3D culture of MSCs enhances cell-cell contact and ECM interactions that increase the secretion of growth factors and boost therapeutic potential (Petrenko et al., 2017; Kouroupis and Correa, 2021). The self-assembly of MSCs into aggregates has important implications for the application of MSCs to cell therapy and tissue regeneration (Sart et al., 2014). In the present study, morphological analysis using SEM revealed that a large number of small vesicles were generated on the MiB surface, which are expected to be extracellular vesicles (EVs). MSC-derived EVs function as cargo that contain and transfer many types of bioactive molecules to target sites, including growth factors, microRNAs, and lipids. Compared with other tissue-derived MSCs, ADMSC-derived EVs have a greater ability to stimulate proliferation and demonstrate therapeutic efficacy in neurological diseases and hepatic regeneration (McLaughlin et al., 2022). Based on reports that the spheroid culture of MSCs enhances the rate of exosome production (Hazrati et al., 2022), our finding of a high protrusion of membrane vesicles on the surface of MiBs suggests that these vesicles originate from an increase in EV production. Further studies are needed to determine which types of membrane vesicles are preferentially produced by MiBs, such as exosomes, microvesicles, or apoptotic bodies, and to investigate the functions of EVs from MiBs.

Using AggreWell400 plates and cell strainers, uniformly sized MiBs were formed with a 104 ± 7.49 μm diameter, which did not affect the viability of MSCs. Moreover, MiBs showed enhanced expression of type I collagen and fibronectin compared with 2D-cultured MSCs. Type I collagen is a key fibrous protein in connective tissue that helps preserve skin elasticity (Salvatore et al., 2021). Although natural collagen has a limited lifespan due to its unstable and easily degraded nature, collagen injections improve skin depressions caused by aging, atrophy, and trauma (Rapaport et al., 1984; Pitaru et al., 2007). Fibronectin binds to integrin receptors and plays an important role in connecting cells with collagen fibers, thereby sealing wounds and regulating neovascularization (Xue and Jackson, 2015). Therefore, the abundant expression of type I collagen and fibronectin in MiBs may improve skin healing when MiBs are applied to atrophic scars or wounds (Xue and Jackson, 2015).

Together with the ECM, growth factors play a crucial role in tissue repair. Bhang et al. showed that MSC spheroids grafted into ischemic limbs ameliorate tissue damage via expression of ECM components and growth factors. According to this previous study, spheroid culture preconditions MSCs to a hypoxic environment, which upregulates hypoxic signaling pathways and activates gene expression involved in angiogenesis and tissue repair. In transplanted MSC spheroids, expression of VEGF, HGF, and HGF-2 are significantly increased, contributing to neovascularization and the regeneration of damaged tissue (Bhang et al., 2011). VEGF induces proliferation of fibroblasts and endothelial cells, neovascularization, and collagen deposition (Johnson and Wilgus, 2014). HGF also contributes to epithelial repair and neovascularization by regulating cell growth and the migration of epithelial and endothelial cells (Borena et al., 2015). MSC spheroids combined with chitosan-hyaluronan membrane or hydrogel also acquired an ability to express high anti-inflammatory cytokines, FGF-1, VEGF, CCL2, CXCR4, and MMP1. When these spheroids combined with gel or membrane were used as wound dressing, accelerated wound healing was observed (Hsu and Hsieh, 2015; Zeng et al., 2022). In our study, we made an additional observation of enhanced expression of VEGF and HGF in MiBs, along with neovascularization within the skin injected with MiBs. It is possible that the expression of growth factors may be play a role in long-term volume retention of MiBs and contribute to tissue repair through angiogenic and regenerative activities. Unlike VEGF and HGF expression, our analysis revealed a decrease in the expression of PDGF and FGF-2 in MiBs. This observation may be related to the absence of active proliferation of ADMSCs in MiBs, as evidenced by DNA content measurements used as a proliferation indicator during the culture period of MiBs.

The treatment of burns should also consider reducing scar formation, minimizing wound infection rates, and establishing growth factors to support angiogenesis. Artificial skin grafts currently used for burn wounds are unable to replicate the intricate structure of the native skin (Phua et al., 2021). Several previous studies have shown that MSCs have beneficial effects on promoting wound healing, inhibiting burn inflammation, and preventing pathological scar formation during burn healing (Shpichka et al., 2019; Wang et al., 2021; Schulman et al., 2022). Therefore, it is expected that ECM and growth factors expressed at high levels in MiB will improve granulation and induce new angiogenesis and re-epithelialization during burn healing. In addition, since MiB does not lose its MSC-specific characteristics, it is anticipated to reduce scar formation by controlling inflammation, which is a characteristic of MSCs.

When MiBs were subcutaneously transplanted into the skin of NSG mice, which have immune tolerance for human cells, a dose-dependent volumizing effect of MiBs was maintained at the injection sites. A report by Rustad et al. on the application of MSC-incorporated pullulan-collagen hydrogel in wound healing shows that single-cell application of MSCs is not sufficient for skin regeneration; instead, functional scaffolds are needed to augment MSC regenerative potential (Rustad et al., 2012). MSCs surrounded by biomimetic scaffolds maintain their stemness properties and secrete high levels of wound healing cytokines compared with regularly cultured cells. In addition, the *in vivo* viability of MSCs within hydrogel scaffolds increases dramatically when injected into wound edges compared with MSC injection alone. Our *in vitro* data also indicates that MiBs express higher levels of VEGF and HGF, which are known to increase angiogenesis, compared to MSCs grown in monolayer culture. Therefore, we expect that MSCs in MiBs will demonstrate superior long-term *in vivo* survival and angiogenic properties due to the stable microenvironment that facilitates cell-to-cell and cell-to-matrix interactions in 3D culture. The long-term persistence of MiBs as dermal fillers suggests the potential for a positive effect on the treatment of atrophic scars. However, direct testing of MiBs on animal models with atrophic scars has not yet been conducted due to a lack of animal model. Therefore, there is a need to develop an experimental animal model to study cell therapy using human cells. As there are currently no xenotransplantation-capable animal models for atrophic scars, an alternative approach could be to test MiBs derived from the same animal. Such testing would provide valuable insights into the potential use of MiBs as a treatment for atrophic scars. In addition, while MiB transplants offer the advantage of maintaining skin volume over a prolonged period, there may still be concerns about tumorigenicity, even with autologous transplantation, since the MiBs are generated from MSCs cultured *in vitro*. To address this issue, it is suggested that a chromosomal examination be conducted beforehand, prior to injecting the MiBs into the skin. Such examination can help to prevent the risk of tumorigenicity associated with the use of MiBs for treating atrophic scars.

MSCs offer advantages for cell therapy due to their low immunogenicity. MSCs express very low levels of major histocompatibility complex (MHC) class I and rare MHC class II, thereby reducing the activation of allogeneic lymphocytes (Le Blanc et al., 2003). However, the presence of MHC class I on MSCs still poses a potential risk of rejection when using allogeneic MSCs. To mitigate the immunogenicity of allogeneic MiBs, solution is to generate MiBs from hypoimmunogenic MSCs that have undergone genetic modifications, such β2-microglobulin (B2M) gene knockout, combined with B2M-HLA-G knock-in (Meshitsuka et al., 2022). This genetic modification protects MSCs from both T cells and NK cells, allowing MiBs composed of the hypoimmunogenic MSCs to evade the attack of immune cells. As a result, these MiBs may achieve long-term survival in allogeneic recipients. Alternatively, to completely eliminate the risk of immune activation through MiB transplantation, MiBs can be generated from autologous MSCs and subsequently engrafted back into the same individual.

In summary, we provide evidence that self-assembled MSC spheroids, called MiBs, could be used as a therapeutic for skin conditions such as severe wounds and skin atrophy. Our findings of high expression of growth factors and ECM components in MiBs and the long-term survival of MiBs after subcutaneous implantation suggest the potential application of MiBs in regenerative medicine for patients with skin conditions such as atrophic scars.

## Data Availability

The original contributions presented in the study are included in the article/Supplementary Material, further inquiries can be directed to the corresponding author.
