# Supplementary material for: Self-assembled adipose-derived mesenchymal stem cells as an extracellular matrix component- and growth factor-enriched filler
Source: Front Cell Dev Biol. 2023 Sep 19;11:1219739. doi: 10.3389/fcell.2023.1219739 (PMC10549996; doi:10.3389/fcell.2023.1219739)
Supplement: Supplementary file 1 [file Table1.DOCX]

Supplementary Material

Self-Assembled Adipose-Derived Mesenchymal Stem Cells as an Extracellular Matrix Component- and Growth Factor-Enriched Filler

Choa Park, Ok-Hee Lee, Jin Ju Park, Jiyoon Yoo, Euna Kwon, Jie-Eun Park, Byeong-Cheol Kang, Dong-Sup Lee, Jaejin Cho*

*** Correspondence:** Jaejin Cho: jcho@snu.ac.kr

# Supplementary Figures and Tables

## Supplementary Figures


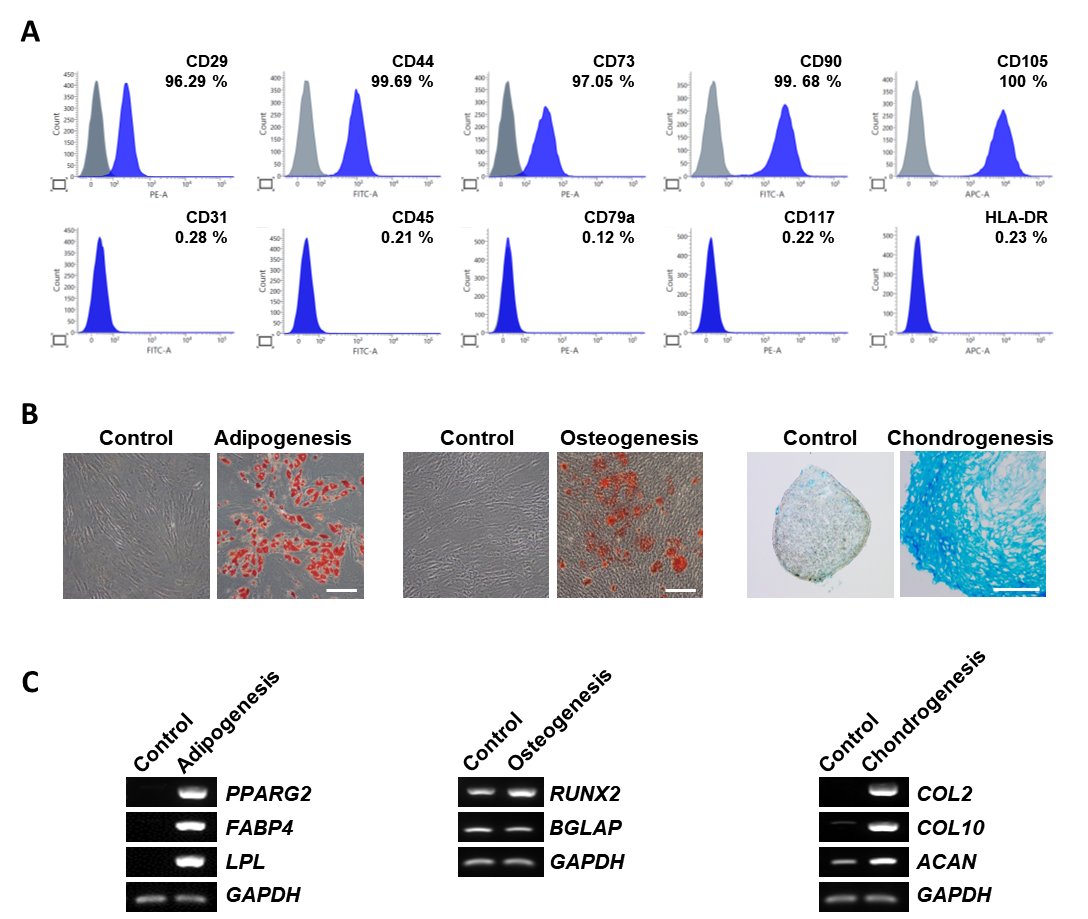


Supplementary Figure S1. Characterization of ADMSCs from donor III. Expressions of positive markers (CD29, CD44, CD73, CD90, and CD105) and negative markers (CD31, CD45, CD79a, CD117, and HLA-DR) of ADMSCs were analyzed using flow cytometry. (B) The differentiation potential of ADMSCs into adipocytes, osteocytes, and chondrocytes was compared with undifferentiated cells by Oil red O, Alizarin Red S, and Alcian Blue staining, respectively. Scale bar, 200 μm. (C) Gene expression of differentiation markers was analyzed by RT-PCR. Total RNA was isolated from undifferentiated and differentiated ADMSCs.


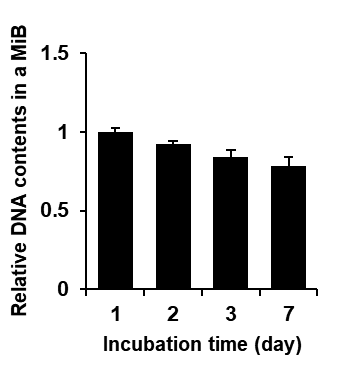


Supplementary Figure S2. Proliferation of ADMSCs in MiBs. DNA content of MiBs was measured to analyze the proliferation of cells in the MiBs. MiBs cultured for 1, 2, 3, and 7 days were harvested and genomic DNA was isolated from a total of 1,200 MiBs. The relative amount of genomic DNA was analyzed using the Quant-iT™ PicoGreen™ dsDNA Assay kit.


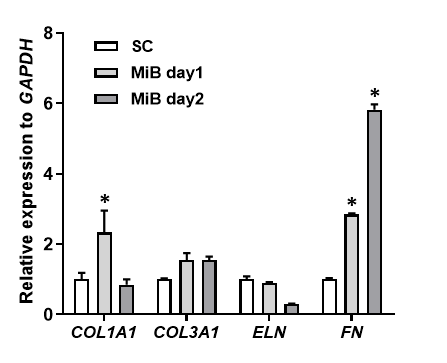


Supplementary Figure S3. Expression of ECM in MiBs at day 1 and 2. Expression of collagen type I (*COL1A1*), collagen type III (*COL3A1*), elastin (*ELN*), and fibronectin (*FN*) was analyzed by real-time RT-PCR. Relative expression was compared with monolayer MSCs (SC). Data represent the means ± SD. * p < 0.05 vs. SC.


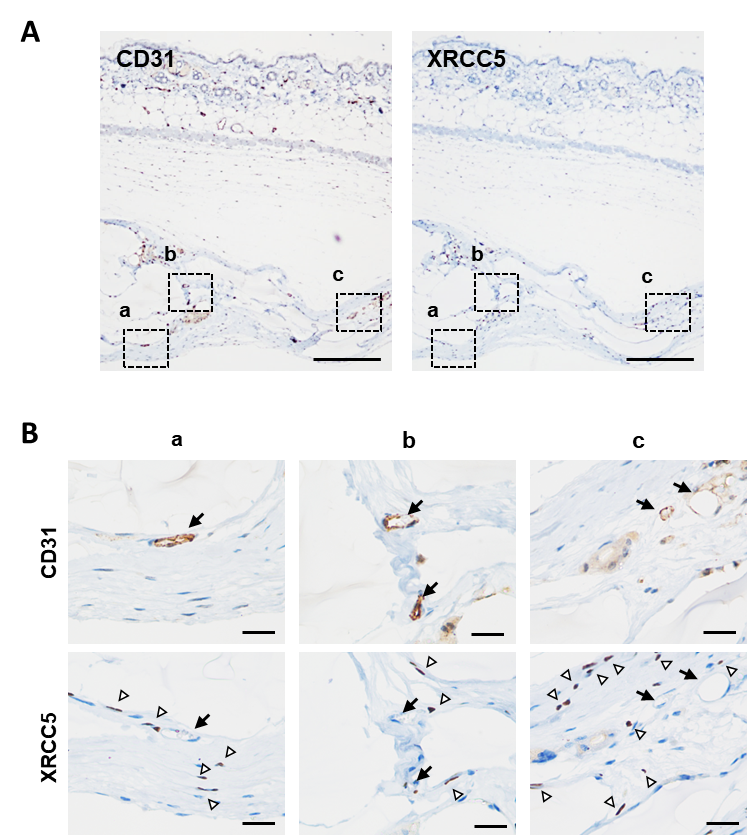


**Supplementary Figure S4**. Vascularization in implanted MiBs. Consecutive sections of mouse skin tissues injected with MiBs were subjected to immunostaining using anti-human/mouse CD31or anti-human XRCC5 (KU80) antibodies. Low magnification images (A) show boxed regions labeled as “a”, “b”, and “c”, which correspond to the high magnification photographs in B. CD31-positive blood vessels are marked with arrows, while XRCC5-positive human cells are indicated with arrowheads. The sections were counterstained with hematoxylin. Scale bars in A represent 200 μm and scale bars in B represent 20 μm, respectively.

## Supplementary Tables

Supplementary Table S1. Primer sets used for Real-time quantitative PCR analysis

| Gene | Sequence | Product size |
| --- | --- | --- |
| GAPDH | Forward; 5’- CCA CTC CTC CAC CTT TGA CG -3’ | 107 bp |
|  | Reverse; 5’- CCA CCA CCC TGT TGC TGT AG -3’ |  |
| VEGFA | Forward; 5’- TGC AGA TTA TGC GGA TCA AAC C -3’ | 81 bp |
|  | Reverse; 5’- TGC ATT CAC ATT TGT TGT GCT GTA G -3’ |  |
| IGF1 | Forward; 5’- TGT GGA GAC AGG GGC TTT TA -3’ | 245 bp |
|  | Reverse; 5’- CCT GCA CTC CCT CTA CTT GC -3’ |  |
| HGF | Forward; 5’- TCA CGA GCA TGA CAT GAC TCC -3’ | 69 bp |
|  | Reverse; 5’- AGC TTA CTT GCA TCT GGT TCC -3’ |  |
| FGF2 | Forward; 5’- TCA AAG GAG TGT GTG CGA AC -3’ | 161 bp |
|  | Reverse; 5’- CAG GGC CAC ATA CCA ACT G -3’ |  |
| PDGFA | Forward; 5’- TCC ATG CCA CTA AGC ATG TG -3’ | 108 bp |
|  | Reverse; 5’- CGT AAA TGA CCG TCC TGG TCT T -3’ |  |

Supplementary Table S2. Primer sets used for RT-PCR

| Gene | Sequence | ℃ (cycle) | Size (bp) |
| --- | --- | --- | --- |
| GAPDH | Forward; 5’- ATG GGG AAG GTG AAG GTC G -3’ | 60 (26) | 119 |
|  | Reverse; 5’- TAA AAG CAG CCC TGG TGA CC -3’ |  |  |
| LPL | Forward; 5’- TAC AGG GCG GCC ACA AGT TTT -3’ | 60 (30) | 299 |
|  | Reverse; 5’- ATG GAG AGC AAA GCC CTG CTC -3’ |  |  |
| FABP4 | Forward; 5’- CAT CAG TGT GAA TGG GGA TG -3’ | 56 (30) | 252 |
|  | Reverse; 5’- GTG GAA GTG ACG CCT TTC AT -3’ |  |  |
| PPARG2 | Forward; 5’- GAC CAC TCC CAC TCC TTT GA -3’ | 60 (30) | 257 |
|  | Reverse; 5’- CGA CAT TCA ATT GCC ATG AG -3’ |  |  |
| RUNX2 | Forward; 5’- TAT GAA AAA CCA AGT AGC AAG GTT C -3’ | 58 (35) | 336 |
|  | Reverse; 5’- GTA ATC TGA CTC TGT CCT TGT GGA T -3’ |  |  |
| BGLPA | Forward; 5’- GTG CAG AGT CCA GCA AAG GT -3’ | 56 (32) | 175 |
|  | Reverse; 5’- CTA GCC AAC TCG TCA CAG TC -3’ |  |  |
| ACAN | Forward; 5’- TTC AGT GGC CTA CCA AGT GGC ATA -3’ | 60 (30) | 165 |
|  | Reverse; 5’- AGC CTG GGT TAC AGA TTC CAC CAA -3’ |  |  |
| COL2 | Forward; 5’- TTT CCC AGG TCA AGA TGG TC -3’ | 56 (29) | 377 |
|  | Reverse; 5’- CTG CAG CAC CTG TCT CAC CA -3’ |  |  |
| COL10 | Forward; 5’- ATG ACC CAA GGA CTG GAA TCT TTA -3’ | 58 (31) | 276 |
|  | Reverse; 5’- CTG AGA AAG AGG AGT GGA CAT AC -3’ |  |  |
